# A Novel Error Detection due to Joint CRC Aided Denoise-and-Forward Network Coding for Two-Way Relay Channels

**DOI:** 10.1155/2014/470324

**Published:** 2014-08-27

**Authors:** Yulun Cheng, Longxiang Yang

**Affiliations:** ^1^Jiangsu Key Lab of Wireless Communications, Nanjing University of Posts & Telecommunications, Nanjing 210003, China; ^2^Key Lab of Broadband Wireless Communication & Sensor Network Technology, Ministry of Education, Nanjing 210003, China

## Abstract

In wireless two-way (TW) relay channels, denoise-and-forward (DNF) network coding (NC) is a promising technique to achieve spectral efficiency. However, unsuccessful detection at relay severely deteriorates the diversity gain, as well as end-to-end pairwise error probability (PEP). To handle this issue, a novel joint cyclic redundancy code (CRC) check method (JCRC) is proposed in this paper by exploiting the property of two NC combined CRC codewords. Firstly, the detection probability bounds of the proposed method are derived to prove its efficiency in evaluating the reliability of NC signals. On the basis of that, three JCRC aided TW DNF NC schemes are proposed, and the corresponding PEP performances are also derived. Numerical results reveal that JCRC aided TW DNF NC has similar PEP comparing with the separate CRC one, while the complexity is reduced to half. Besides, it demonstrates that the proposed schemes outperform the conventional one with log-likelihood ratio threshold.

## 1. Introduction

Although the capacity bounds of two-way (TW) communication channel were first studied and calculated in the 1960s [1], various cooperative strategies have been extended to transmit over TW relay channels [2–6] only since the advent of network coding (NC) [7, 8] in recent years. In general, TW NC is consisted of multiaccess (MA) and broadcasting (BC) phases, and in the existing literatures, there are three main NC schemes that were studied in MA phase, which are XOR NC [8], amplify-and-forward (ANF) NC [9], and denoise-and-forward (DNF) NC [10]. Among these schemes, DNF is one of the most promising ones due to its very high spectral efficiency, since the TW communication can be accomplished within 2 time slots (TS), while 3 TS are required for XOR NC and ANF NC. This merit makes DNF NC very attractive in both industrial and academic areas in recent years. Despite the potential advantage, the performance loss caused by error propagation is also apparent. As shown in [11], fixed DNF without error check can offer almost no diversity gain in large signal-noise-ratio (SNR) region, while in [12, 13], the results similarly indicate that the signal reliability in MA phase dominates the performance of the whole system and any error occurred will be severely propagated among all the cooperative users. Hence, to mitigate error propagation effectively, DNF is always employed jointly with adaptive signal reliability check at relay. For example, [11, 14] utilize the metric based on SNR or square amplitude of channel gain to represent signal reliability. For accuracy improvement, [15] proposes the log-likelihood ratio- (LLR-) based threshold to replace the SNR-based one in the cooperative multiple access channels. In [12], this type of threshold is introduced into TW NC to evaluate the reliability of network coded bits, named as NC bit threshold. Besides these uncoded methods, [16, 17] employ cyclic redundancy code (CRC) check at relay for error bit identification. In all the above schemes, the detection or check is always operated separately; in other words, each signal from different cooperative users is checked independently, and the relay can decode and forward these signals only when all of them pass the check. Different from these former works, [18] first proposes joint-CRC (JCRC) check in space-time-block coded cooperation system, in which CRC check is applied to the mixed signals. However, the performance evaluation of this type check is only presented by simulations individually, not by theoretical derivation, nor compared with separate CRC (SCRC) check either. Besides, the impact of JCRC check on pairwise error probability (PEP) performance is also not analyzed theoretically. These two points motivate the work presented in this paper.

The contribution of this paper is threefold.

- Several theoretical bounds are derived and compared with SCRC check to evaluate the effectiveness of JCRC check in TW relay channels.
- 3 JCRC aided DNF NC schemes are proposed, and with the obtained bounds mentioned above, the PEP performances of the proposed schemes are also derived.
- The effectiveness of the derived bounds and the proposed schemes are confirmed by the simulation results.

The rest of this paper is organized as follows.  Section 2 presents the system model and the theoretical bounds of detection reliability probability for JCRC check.  Section 3 describes 3 JCRC aided DNF schemes, while the corresponding PEP analysis is presented in Section 4. Simulation results are shown in Section 5. Finally, Section 6 concludes the paper.

## 2. System Model

### 2.1. System Model

**Figure 1:**
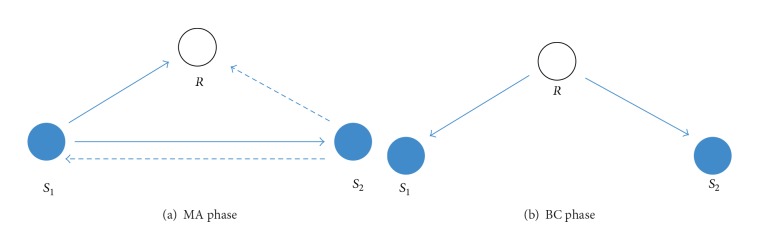
System model of TW relay channel.

We consider a TW relay channel, where two source nodes *S*_1_ and *S*_2_ exchange information, with the aid of a relay node *R* in between them, as is shown in Figure 1. All the nodes are equipped with signal antenna and communicate over the same frequency band. To meet the practice situation, we assume that each node cannot transmit and receive signal at the same time, while time division multiplexing (TDM) is employed for channel access.

As depicted in Figures 1(a) and 1(b), the whole transmission consists of MA and BC phases. In MA phase, each source node converts its own information into the CRC codeword and broadcasts it to each other during the first 2 TS; so the received signals at both relay and source nodes can be written as

(1) $$\begin{array}{c} r_{iR}=\sqrt{E_{s}}h_{iR}\cdot s_{i}+n_{R},\;i=1,2, \end{array}$$

(2) $$\begin{array}{c} r_{ij}=\sqrt{E_{s}}h_{ij}\cdot s_{i}+n_{j},\;i,j\in \left\{1,2\right\},\;\;i\neq j, \end{array}$$

where **r**_*iR*_ and **r**_*ij*_ are the received signals at *R* and *S*_*j*_, respectively, from *S*_*i*_. It is assumed that *S*_1_ and *S*_2_ employ and transmit the same length of (*n*, *k*) CRC codeword, so **r**_*iR*_ and **r**_*ij*_ are both *n*-length vectors. **s**_*i*_ is the CRC codeword transmitted by *S*_*i*_, and it can be expressed as

(3) $$\begin{array}{c} s_{i}={\left[s_{i}^{\left(1\right)},s_{i}^{\left(2\right)}\cdots s_{i}^{\left(n\right)}\right]}^{T},\;s_{i}^{\left(j\right)}\in \left\{+1,-1\right\}, \end{array}$$

where binary phase shift keying (BPSK) modulation is assumed. Let **h**_*iR*_ capture the effects of frequency nonselective multipath fading between *S*_*i*_ and *R*, and it can be written as

(4) $$\begin{array}{c} h_{iR}={\left[\left|h^{\left(1\right)}\right|e^{j\theta^{\left(1\right)}},\left|h^{\left(2\right)}\right|e^{j\theta^{\left(2\right)}},\ldots ,\left|h^{\left(n\right)}\right|e^{j\theta^{\left(n\right)}}\right]}^{T}, \end{array}$$

where *h* ^(*i*)^ is modeled as zero-mean, independent, and circular-symmetric complex Gaussian random variable with variances *σ*_*iR*_^2^ and *θ* ^(*i*)^ is the corresponding phase variable with uniform distribution. It should be noted |*h* ^(*i*)^|^2^ follows exponential distribution, where the distribution parameter *λ*_*iR*_ = 1/*σ*_*iR*_^2^, and is dependent on the distance between *S*_*i*_ and *R*. Similarly, **h**_*ij*_ is the channel gain between *S*_*i*_ and *S*_*j*_, and we assume that the uplink and downlink channels between two nodes are reciprocal; that is, **h**_*ij*_ = **h**_*ji*_. Let **n**_*R*_ and **n**_*j*_ capture the effects of receiver noise at *R* and *S*_*j*_, respectively, and are modeled as zero-mean complex Gaussian random sequences with variance *N*_0_. We denote *E*_*s*_ as the average transmitting power, and *E*_*s*_/*N*_0_ represents the signal-noise-ratio (SNR) throughout this paper. After MA phase, relay *R* tries to decode each **s**_*i*_ from **r**_*iR*_ with maximum likelihood detection (MLD) as

(5) s^i=arg min⁡si∈W|Re(hiR∗·riR)−Es|hiR|2·si|, i=1,2,

in which ${\hat{s}}_{i}$ is the estimation of **s**_*i*_ and *W* is the transmit codebook. *Re*(*z*) denotes the real part of a complex number *z*, and (·)* indicates the complex conjugation.

Based on that, SCRC is employed to identify error bits; that is, each ${\hat{s}}_{i}$ is checked by CRC function and generate the corresponding check remainder $c({\hat{s}}_{i})$. It should be noted that according to the CRC check principle [19], $c({\hat{s}}_{i})$ is generated by the division of ${\hat{s}}_{i}$ and primitive polynomial, and if $c({\hat{s}}_{i})=0$, ${\hat{s}}_{i}$ can be considered as error-free with high probability. Hence, when both $c({\hat{s}}_{i})=0$, *i* = 1,2, relay *R* will combine and forward ${\hat{s}}_{1}$ and ${\hat{s}}_{2}$ as $s_{R}=f({\hat{s}}_{1},{\hat{s}}_{2})$, where *f*(·) is the NC mapping function. Although there exist different protocols for NC mapping, including bit-level XOR [8] and electromagnetic wave-level remapping [10], the actual effects are all equivalent to mod-2 operation in bit-level; that is, $s_{R}={\hat{s}}_{1}⊕{\hat{s}}_{2}$.

In BC phase, *R* broadcasts **s**_*R*_ to both *S*_1_ and *S*_2_; so the received signals at *S*_*i*_ can be expressed as

(6) $$\begin{array}{c} r_{Ri}=\sqrt{E_{s}}h_{Ri}\cdot s_{R}+n_{i},\;i=1,2. \end{array}$$

We assume that perfect channel state information (CSI) can be achieved at all the receivers; so *S*_*i*_ first decode **s**_*R*_ form **r**_*Ri*_ through MLD as

(7) s^R=arg min⁡si∈W|Re(hRi∗·rRi)−Es|hRi|2·si|,

in which ${\hat{s}}_{R}$ is estimation of **s**_*R*_. After that, maximal ratio combination (MRC) is employed to estimate **s**_*j*_ as

(8) s^j=arg min⁡si∈  W|Re(hRi∗·rRi·f−1(s^R,si)+hij∗·rij)     −Es(|hRi|2+|hij|2)·si|,

where *f* ^−1^(·) is the NC inverse mapping function, while **s**_*i*_ is preknown *S*_*i*_. Besides, if $c({\hat{s}}_{i})\neq 0$, *R* will keep silent in BC phase, and **s**_*j*_ is estimated by substituting **r**_*Ri*_ = 0 into (8).

### 2.2. Theoretical Bounds of JCRC Check

This paper aims to introduce JCRC check into TW DNF NC for effective error mitigation. Firstly, the equivalence of JCRC and SCRC check in TW relay channel is proved by the following theorem.

**Theorem 2.2 I.**

In the above TW relay model, as SNR increases, there is $\mathrm{Pr}(c({\hat{s}}_{R})=0)\approx \mathrm{Pr}(c({\hat{s}}_{1})=0)\cdot \mathrm{Pr}(c({\hat{s}}_{2})=0)\approx 1$.

**Proof**

According to the property of CRC codeword [19], there is **s**_*R*_ = **s**_1_ ⊕ **s**_2_ ∈ *W* [16] when **s**_1_, **s**_2_ ∈ *W*. Hence, it can be deduced that $c({\hat{s}}_{R})=c({\hat{s}}_{1}⊕{\hat{s}}_{2})=c({\hat{s}}_{1})⊕c({\hat{s}}_{2})$. The above equation indicates that $c({\hat{s}}_{R})=0$ only when *a* happens, where *a* ∈ *A*, and the set *A* can be written as

(9) $$\begin{array}{c} A=\left\{c\left({\hat{s}}_{1}\right)=0\right\}\cap \left\{c\left({\hat{s}}_{2}=0\right)\right\}\cup \overset{n}{\underset{i=1}{\sum }}\left\{{\hat{s}}_{1}^{\left(i\right)}\neq s_{1}^{\left(i\right)}\right\}\cap \left\{{\hat{s}}_{2}^{\left(i\right)}\neq s_{2}^{\left(i\right)}\right\}. \end{array}$$

Let *γ*_*m*_ denote the error bit number in ${\hat{s}}_{m}$, *m* = 1,2; then the detection probability $\mathrm{Pr}(c({\hat{s}}_{m})=0)$ can be bounded as

(10) Pr⁡(c(s^m)=0)=Pr⁡(c(s^m)=0,γm≠0) +Pr⁡(c(s^m)=0,γm=0)≥Pr⁡(c(s^m)=0,γm=0).

According to Theorems 5–11 in [19], $\mathrm{Pr}(c({\hat{s}}_{m})=0,\gamma_{m}=0)$ can be further bounded as

(11) Pr⁡(c(s^m)=0,γm≠0)≤∑i=1n−k−1Pr⁡⁡(γm=i) +∑j=n−knPr⁡(γm=j)·(1−2−(n−k−1)).

We assume that $P_{bm}=\mathrm{Pr}({\hat{s}}_{m}^{\left(i\right)}\neq s_{m}^{\left(i\right)})$ denotes the average bit error probability (BEP) of BPSK symbol over Rayleigh fading channel plus zero-mean unit-variance additive white Gaussian noise (AWGN), and it is easily deduced as [20, equation (14.3-7)]

(12) $$\begin{array}{c} P_{bm}=\frac{\left(1-\sqrt{\left(E_{s}/\lambda_{m}N_{0}\right)/\left(1+E_{s}/\lambda_{m}N_{0}\right)}\right)}{2}, \end{array}$$

in which *λ*_*m*_ = 1/*σ*_*mR*_^2^. Hence, it can be derived that

(13) $$\begin{array}{c} \mathrm{Pr}\left(\gamma_{m}=i\right)=\left(\begin{array}{c} n\\ i \end{array}\right){\left(P_{bm}\right)}^{i}\times {\left(1-P_{bm}\right)}^{n-i},\;i=1\ldots n,\\ \mathrm{Pr}\left(c\left({\hat{s}}_{m}\right)=0,\gamma_{m}=0\right)={\left(1-P_{bm}\right)}^{n}. \end{array}$$

Substituting (11)–(13) into (10), the upper and lower bounds of $\mathrm{Pr}(c({\hat{s}}_{m})=0)$ can be obtained as

(14) $$\begin{array}{c} B_{1}\leq \mathrm{Pr}\left(c\left({\hat{s}}_{m}\right)=0\right)\leq B_{2}, \end{array}$$

in which

(15) $$\begin{array}{c} B_{1}={\left(\frac{1}{2}+\frac{\sqrt{(E_{s}/\lambda_{m}N_{0})/\left(1+E_{s}/\lambda_{m}N_{0}\right)}}{2}\right)}^{n}, \end{array}$$

(16) B2=B1+∑i=1n−k−1(ni)(Pbm)i×(1−Pbm)n−i +∑j=n−kn(nj)(Pbm)j×(1−Pbm)n−j×(1−2−(n−k−1)).

With the above results, the upper and lower bounds of detection reliability for SCRC check can be calculated as

(17) $$\begin{array}{c} \overset{2}{\underset{m=1}{\prod }}B_{1}\left(m\right)\leq \mathrm{Pr}\left(c\left({\hat{s}}_{1}\right)=0,c\left({\hat{s}}_{2}\right)=0\right)\leq \overset{2}{\underset{m=1}{\prod }}B_{2}\left(m\right), \end{array}$$

in which *B*_*i*_ is written as the function form *B*_*i*_(*m*), *i* = 1,2, and can be calculated by (15).

For JCRC check, according to the definition of the set *A* and (10), $\mathrm{Pr}(c({\hat{s}}_{R})=0)$ can be calculated as

(18) Pr⁡(c(s^R)=0)=Pr⁡(c(s^1)=0,c(s^2)=0) +Pr⁡(∑i=1n{s^1(i)≠s1(i)}∩{s^2(i)≠s2(i)}),

(19) Pr⁡(∑i=1n{s^1(i)≠s1(i)}∩{s^2(i)≠s2(i)}) =∑i=1nPr⁡(γ1=i)×(Pb2)i×(1−Pb2)n−i.

Note that (19) is due to the fact that, when the error bits coincidentally occur in the same position of ${\hat{s}}_{1}$ and ${\hat{s}}_{2}$, it will not be identified by JCRC check, because the mod-2 operation of two error bits can still obtain the correct bit. Hence, (19) indicates that JCRC will bring more NC opportunities compared with SCRC check. Substituting (12), (17)–(19) into (18), the detection reliability bounds of JCRC can be calculated as

(20) $$\begin{array}{c} \overset{2}{\underset{m=1}{\prod }}B_{3}\left(m\right)\leq \mathrm{Pr}\left(c\left({\hat{s}}_{R}\right)=0\right)\leq \overset{2}{\underset{m=1}{\prod }}B_{4}\left(m\right), \end{array}$$

in which

(21) Bm=Bm−2+∑i=1nPr⁡(γ1=i)×(Pb2)i×(1−Pb2)n−i,m=3,4.

Since there is always *B*_*m*_ > *B*_*m*−2_, it can be easily concluded that

(22) $$\begin{array}{c} \mathrm{Pr}\left(c\left({\hat{s}}_{R}\right)=0\right)>\mathrm{Pr}\left(c\left({\hat{s}}_{1}\right)=0\right)\times \mathrm{Pr}\left(c\left({\hat{s}}_{2}\right)=0\right). \end{array}$$

According to (18), it can be seen that as SNR increases, $\mathrm{Pr}(c({\hat{s}}_{R})=0)\approx \mathrm{Pr}(c({\hat{s}}_{1})=0)\cdot \mathrm{Pr}(c({\hat{s}}_{2})=0)$ because (19) tends to 0. At the same time, *P*_*bm*_ tends to 0, which indicates that *B*_*m*_ tends to *B*_*m*−2_ while *B*_1_ tends to 1; thus, Theorem I is obtained.

## 3. JCRC Aided DNF NC Protocol

On the basis of Theorem I, 3 JCRC aided DNF NC schemes are proposed in TW relay channels in this section.

### 3.1. 3 TS Decode-JCRC-Forward (3T JCRC) 

In the first 2 TS, ${\hat{s}}_{1}$ and ${\hat{s}}_{2}$ are gotten at *R* from coherent reception of **r**_1*R*_ and **r**_2*R*_, respectively. Then NC is accomplished at bit-level; that is, $s_{R}={\hat{s}}_{1}⊕{\hat{s}}_{2}$. After that, **s**_*R*_ is checked by JCRC, and if *c*(**s**_*R*_) = 0, *R* will forward **s**_*R*_ to *S*_1_ and *S*_2_ at BC phase. Otherwise, *R* keeps silent. Note that there are two main differences between this scheme and former TW denoise-XOR-forward CRC NC [8].

- XOR is operated before CRC check in this scheme while in the former one, the bits are XORed only when both the codewords pass the CRC check.
- Only once JCRC check is operated to **s**_*R*_ in this scheme, while in the former one, 2 SCRC checks are required for ${\hat{s}}_{1}$ and ${\hat{s}}_{2}$, respectively.

### 3.2. 2 TS Decode-JCRC-Forward with Phase Synchronization (2T JCRC-P)

It is proposed in [10] that the transmitted signals are precoded when CSI is known in the source nodes, so that it allows the direct merger of two signals from the source nodes in the form of electromagnetic waves at relay simultaneously. Relay then remaps the merged signals into one NC signal and broadcast it in BC phase. Only 2 TS are consumed for TW communication by this so-called physical layer NC (PNC). This paper introduces JCRC check into PNC system to judge the quality of the remapped signals. Assuming that only the phase synchronization is operated in MA phase, the received and remapped signals at relay can be written as

(23) $$\begin{array}{c} r=\sqrt{E_{s}}\left(\left|h_{1R}\right|\cdot s_{1}+\left|h_{2R}\right|\cdot s_{2}\right)+n_{R}, \end{array}$$

(24) $$\begin{array}{c} s_{R}=f\left(r\right), \end{array}$$

in which

(25) $$\begin{array}{c} f\left(x^{\left(i\right)}\right)=\{\begin{array}{cc} -1, & \text{if}\;\left|x^{\left(i\right)}\right|>\sqrt{E_{s}}\max\left\{\left|h_{1R}^{\left(i\right)}\right|,\left|h_{2R}^{\left(i\right)}\right|\right\}\\ 1, & \text{if}\;\left|x^{\left(i\right)}\right|<\sqrt{E_{s}}\max\left\{\left|h_{1R}^{\left(i\right)}\right|,\left|h_{2R}^{\left(i\right)}\right|\right\}. \end{array} \end{array}$$

After that, the remapped signal **s**_*R*_ is detected by JCRC check, and if *c*(**s**_*R*_) = 0, relay will forward **s**_*R*_ at BC phase.

### 3.3. 2 TS Decode-JCRC-Forward with Phase and Amplitude Synchronization (2T JCRC-PA)

Assuming both phase and amplitude synchronization are ideally achieved at *S*_1_ and *S*_2_ in MA phase, the received and remapped signals at relay can be expressed as

(26) $$\begin{array}{c} r=\sqrt{E_{s}}\left(s_{1}+s_{2}\right)+n_{R}. \end{array}$$

Note that this is the special case of (23) when |*h*_1*R*_^(*i*)^ | = |*h*_2*R*_^(*i*)^ | = 1, so the remapping operation is similar to the scheme mentioned above, and *f*(·) in (25) becomes the normal BPSK demodulation.

## 4. PEP Analysis 

In this section, to evaluate the performance of the proposed schemes, we derive the PEP with the obtained theoretical bounds in Theorem I.

Let *d*_min⁡_ denote the minimal Hamming distance of a (*n*, *k*) system CRC codebook, and according to the principle of error correction [19], any received codeword that belongs to the codebook is able to correct at most ⌊*d*_min⁡_ − 1⌋/2 random error bits itself. In general, the PEP of the denoise-JCRC-forward scheme can be written as follows:

(27) Pe=Pr⁡(c(s^R)=0)×Pr⁡(γMRC>⌊dmin⁡−1⌋2) +Pr⁡(c(s^R)≠0)×Pr⁡(γD>⌊dmin⁡−1⌋2)≈Pr⁡(c(s^R)=0)×Pr⁡(γMRC>⌊dmin⁡−1⌋2),

in which *γ*_MRC_ and *γ*_*D*_ denote the error bit number of the signal estimation at *S*_*i*_ when *R* broadcasts **s**_*R*_ and keeps silent, respectively. The approximation in (27) is due to the fact that *Pr*⁡(*c*(**s**_*R*_ ≠ 0)) ≈ 0 when SNR increases, which has been proved in Theorem I. *Pr*⁡(*γ*_MRC_ > ⌊*d*_min⁡_ − 1⌋/2) can be expressed as

(28) $$\begin{array}{c} \mathrm{Pr}\left(\gamma_{\text{MRC}}>\frac{\left\lfloor d_{\min}-1\right\rfloor }{2}\right)=\overset{n}{\underset{i=\left\lfloor d_{\min}-1\right\rfloor /2}{\sum }}\left(\begin{array}{c} n\\ i \end{array}\right){\left(P_{R}\right)}^{i}{\left(1-P_{R}\right)}^{n-i}, \end{array}$$

in which the BEP *P*_*R*_ can be obtained with the results deduced by [15] as

(29) PR={(1−(λsRN0/Es)/(1+λsRN0/Es))2(2+(λsRN0/Es)/(1+λsRN0/Es))4,  if  λsD=λsRλsD2(λsD−λsR)[1−11+λsDN0/Es]−λsR2(λsD−λsR)[1−11+λsRN0/Es],if  λsD≠λsR,

in which *λ*_*sD*_ and *λ*_*sR*_ are the distribution parameters of **h**_*iR*_ and **h**_*ij*_, respectively.

By comparing (1) and (23), it can be concluded that 2T JCRC-P and 3T JCRC will achieve similar PEP, because the only difference between these two schemes is the variance of receiver noise at *R*. Since $\mathrm{Pr}(c({\hat{s}}_{R})=0)$ can be approximated by the lower bound of (17), the PEP of these two schemes can be calculated by substituting (17), (28), and (29) into (27):

(30) Pe 3T JCRC(2T JCRC-P)⁡ ≈(12+(Es/N0λm)/(1+Es/N0λm)2)n  ×∑i=⌊dmin⁡−1⌋/2n(ni)(PR)i(1−PR)n−i.

For 2T JCRC-PA, $\mathrm{Pr}(c({\hat{s}}_{R})=0)$ can be approximated by the lower bound of (20), in which *P*_*bm*_ can be written according to the results of [21] as

(31) $$\begin{array}{c} P_{bm}=\frac{1}{2\pi }\left(\overset{\pi /2}{\underset{0}{\int }}3e^{-(E_{s}/2\sigma^{2}N_{0}\sin\theta )}-e^{-(9E_{s}/2\sigma^{2}N_{0}\sin\theta )}\right)d\theta . \end{array}$$

Hence, the corresponding PEP can be calculated as

(32) Pe 2T JCRC-PA ≈[12π(∫0π/22π−3e−(Es/2σ2N0sinθ)      +e−(9Es/2σ2N0sinθ))dθ]n  ×∑i=⌊dmin⁡−1⌋/2n(ni)(PR)i(1−PR)n−i.

## 5. Simulation Results

In this section, the Monte-Carlo simulation is employed to estimate the detection probability of JCRC check and the PEP performance of the three proposed schemes. Besides, the performance of LLR-based TW NC algorithm in [12] is also simulated as the baseline. The simulation parameters are as follows: the simulation length involved in all points is fixed at 10 million BPSK-modulated codewords. *S*_1_, *S*_2_, and *R* are all equipped with single antenna. SNR denotes *E*_*s*_/*N*_0_, and we assume that all the nodes transmit with the same power. The path loss effects are modeled and parameterized as follows: the reference distance is *d*_0_, and the channel gain parameter can be calculated as *λ*_*i*_ = *λ*_0_ · (*d*_*i*_/*d*_0_)^*γ*^, in which *γ* = 3.5 (typical urban). The system model is as shown in Figure 1, and we assume that *λ*_1*R*_ = *λ*_2*R*_ = 1, *λ*_12_ = 2. A (7, 4) system CRC code is employed, which is generated by the origin polynomial with the minimum Hamming distance *d*_min⁡_ = 3. MLD is applied to estimate the signals at all the terminals. Since, in [12], LLR threshold is bit-oriented, the information rate is fixed as 1 bit/s/channel, and so its PEP equals the BEP. In all the simulations, the LLR threshold is set by the target BEP of 1%, and LLR-PA denotes that the checked signals are perfectly synchronized with both phase and amplitude.

**Figure 2:**
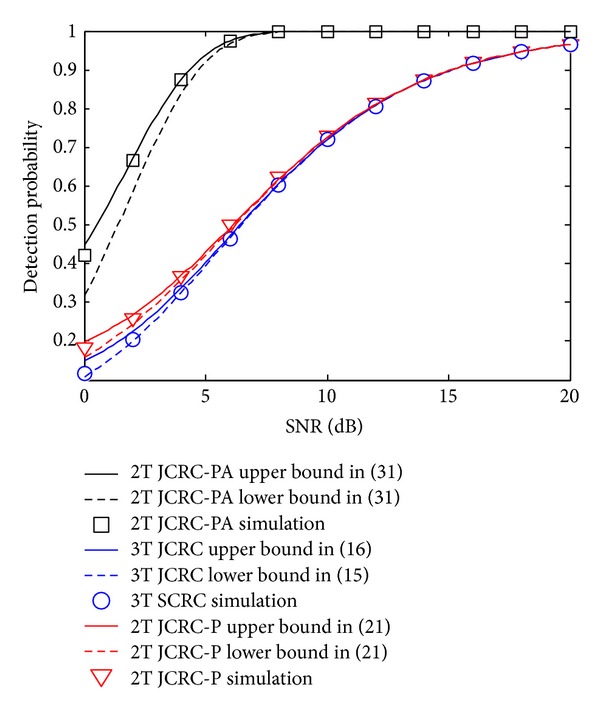
Detection probability comparison of JCRC and SCRC.

Figure 2 depicts the detection probability comparisons of JCRC and SCRC check at *R*. It is verified from the figure that the derived bounds in (15), (16), and (21) coincide well with the simulation results. Compared with SCRC, it can be seen that there is slight higher detection probability for JCRC in the regions of low and medium SNR. This is caused by the more loose restrictive conditions of JCRC check and that brings more opportunities to the relay to code and forward the received signals. However, this merit is not very obvious and gradually disappears when SNR increases. Compared with 3T JCRC and 2T JCRC-P, a distinct advantage can be achieved for 2T JCRC-PA in all SNR regions. As shown in the figure, when SNR > 6 dB, the detection probability is close to 1. This is mainly caused by two reasons: (1) with the phase and amplitude compensation, the PNC signals at *R* are of good quality. (2) JCRC check matches the PNC signals well. This advantage enables 2T JCRC-PA to have more opportunities to exploit relay channel to obtain superior PEP performance.

**Figure 3:**
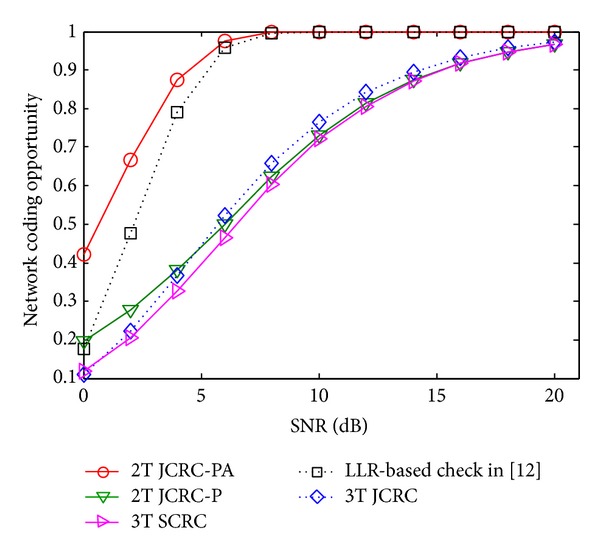
NC opportunities comparison.

Figure 3 shows the NC opportunities comparison of the schemes at *R*, in which NC opportunity is defined as the probability that $c({\hat{s}}_{R})=0$, because the relay *R* is able to combine the received signals through NC if that happens. As is depicted, when SNR increases, the NC opportunities tend to 1 for all the compared schemes, among which 2T JCRC-PA is optimal. Besides, the metric is also close to 95% for LLR-based check, when SNR > 6 dB. The results show that 2T JCRC-PA is superior to LLR-based check in all SNR regions, which indicates that 2T JCRC-PA is able to fully exploit the relay channel to improve PEP performance. Moreover, the performance gaps are not obvious among 3T SCRC, 3T JCRC, and 2T JCRC-P, which indicates that these schemes will have similar opportunity to exploit relay channel through NC. However, it cannot be inferred that they achieve similar PEP performance, which will be discussed as follows.

**Figure 4:**
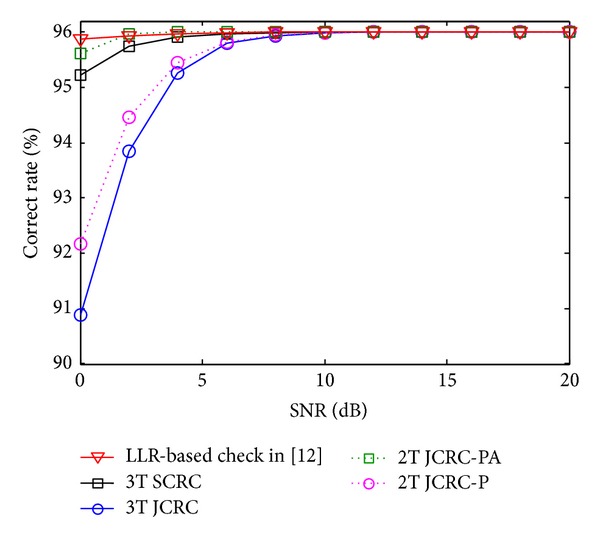
Correct rate comparison of the signals that have successfully passed the check.

Figure 4 shows the correct rate of the signals which has successfully passed the check. As can be seen in the figure, CRC-based and LLR-based checks are both effective measures to evaluate the reliability of the signals, because when SNR > 4 dB, all the compared check accuracies tend to 96%, and even when SNR = 0 dB, most of the compared accuracies are higher than 95%, except 3T JCRC and 2T JCRC-P. In general, CRC-based checks are superior to LLR-based one, but the performance gap is slight. In addition, in low SNR region, 3T SCRC and 2T JCRC-P are less efficient than other schemes, which indicates that JCRC is inferior to SCRC in low SNR region. This result indicates that although JCRC can enable the relay to obtain more opportunities to code and forward received signals, the relative low correct rate will degenerate the PEP performance in low SNR region, which make the gain less obvious compared with SCRC.

**Figure 5:**
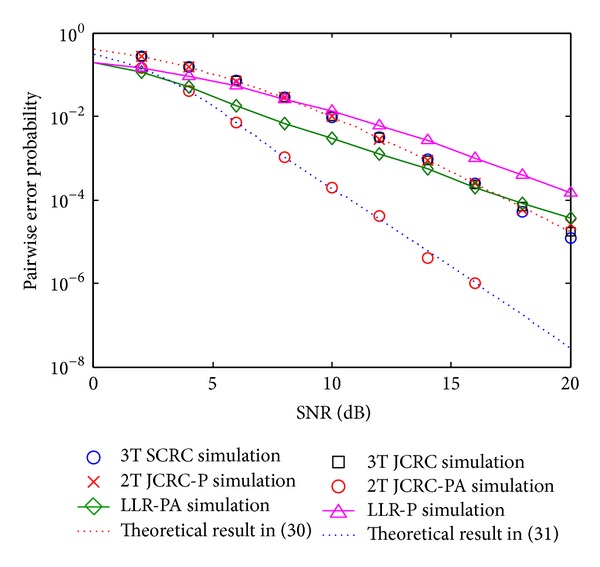
PEP comparisons between theoretical results and simulations.

Figure 5 depicts the PEP comparisons of the proposed schemes. It can be observed that the theoretical results derived by (30) and (31) correspond well with the simulations, which verify the effectiveness of Theorem I and the analysis presented in Section 4. The figure shows that 2T JCRC-PA outperforms the other compared schemes, which also corresponds well with the results in Figure 3, because 2T JCRC-PA enables the relay to obtain more opportunities to exploit the relay channel. Besides, it can be observed that the PEP gap is not obvious among 3T SCRC, 3T JCRC, and 2T JCRC-P. On the one hand, this result verifies the effectiveness of JCRC check; on the other hand, it also confirms that the PEP of 2T JCRC-P and 3T JCRC can be approximated as (30). Moreover, the figure also indicates that 2T JCRC-P outperforms LLR-P scheme in [12] with only phase synchronization, while 2T JCRC-PA is superior to LLR-PA. In the former case, the SNR gain is 2 dB when the target PEP is 10^−3^, while in the latter case, it grows to 4 dB. This gain growth is due to the error correction of CRC code, because for the simulated (7, 4) CRC, any single random error in the codeword can be recovered, which cannot be obtained for LLR check [12]. This advantage makes JCRC check more efficient to mitigate error propagation. Meanwhile, it should be noted that, for the proposed schemes, the spectral efficiency is sacrificed as the cost since the information rate = 4/7 bit/s/channel, while for LLR-based scheme, the metric equals 1 bit/s/channel.

## 6. Conclusion

In this paper, we introduced JCRC check into the TW DNF NC system to evaluate the reliability of NC signals. Firstly, the detection probability bounds of JCRC are theoretically derived to prove its effectiveness. On the basis of that, three JCRC aided TW DNF NC schemes are proposed, and the corresponding PEP performances are also derived. Theoretical and simulation results indicate that the proposed JCRC schemes outperform conventional SCRC check in TW relay communications with only half complexity, because JCRC enables the relay node to have more opportunities to exploit the relay channel, which is helpful for PEP improvement. Moreover, it also shows that JCRC check is superior to the LLR-based scheme [12] in terms of PEP, at the cost of spectral efficiency. In future work, it is of significance to introduce JCRC check into cooperative multiple access channels for error mitigation.

## References

[1] Shannon C Two-way communication channels.

[2] Ding Z, Wang T, Peng M, Wang W, Leung KK (2011). On the design of network coding for multiple two-way relaying channels. *IEEE Transactions on Wireless Communications*.

[3] Louie RHY, Li Y, Vucetic B Performance analysis of physical layer network coding in two-way relay channels.

[4] Louie RHY, Li Y, Vucetic B (2010). Practical physical layer network coding for two-way relay channels: performance analysis and comparison. *IEEE Transactions on Wireless Communications*.

[5] Li Y, Louie RHY, Vucetic B (2010). Relay selection with network coding in two-way relay channels. *IEEE Transactions on Vehicular Technology*.

[6] Zhou QF, Li Y, Lau FCM, Vucetic B (2010). Decode-and-forward two-way relaying with network coding and opportunistic relay selection. *IEEE Transactions on Communications*.

[7] Ahlswede R, Cai N, Li SR, Yeung RW (2000). Network information flow. *IEEE Transactions on Information Theory*.

[8] Chen Y, Kishore S, Li J Wireless diversity through network coding.

[9] Popovski P, Yomo H (2007). Wireless network coding by amplify-and-forward for bi-directional traffic flows. *IEEE Communications Letters*.

[10] Zhang S, Liew S, Lam P Hot topic: physical-layer network coding.

[11] Laneman JN, Tse DNC, Wornell GW (2004). Cooperative diversity in wireless networks: efficient protocols and outage behavior. *IEEE Transactions on Information Theory*.

[12] Nguyen SLH, Ghrayeb A, Al-Habian G, Hasna M (2010). Mitigating error propagation in two-way relay channels with network coding. *IEEE Transactions on Wireless Communications*.

[13] Krikidis I (2010). Relay selection for two-way relay channels with MABC DF: a diversity perspective. *IEEE Transactions on Vehicular Technology*.

[14] Bletsas A, Khisti A, Reed DP, Lippman A (2006). A simple cooperative diversity method based on network path selection. *IEEE Journal on Selected Areas in Communications*.

[15] Khuong HV, Kong HY (2006). LLR-based decode-and-forward protocol for relay networks and closed-form BER expressions. *IEICE Transactions on Fundamentals of Electronics, Communications and Computer Sciences*.

[16] Sadek AK, Su W, Liu KJR (2007). Multinode cooperative communications in wireless networks. *IEEE Transactions on Signal Processing*.

[17] Elfituri M, Hamouda W, Ghrayeb A (2009). A convolutional-based distributed coded cooperation scheme for relay channels. *IEEE Transactions on Vehicular Technology*.

[18] Zhu K, Burr AG Relay selection aided distributed space-time block code for two-way relay channel with physical-layer network coding.

[19] Lin S, Costello D (2004). *Error Control Coding*.

[20] Proakis J (2001). *Digital Communications*.

[21] Lu K, Fu S, Qian Y, Chen H SER performance analysis for physical layer network coding over AWGN channels.

